# Use of Commercial Claims Data for Evaluating Trends in Lyme Disease Diagnoses, United States, 2010–2018

**DOI:** 10.3201/eid2702.202728

**Published:** 2021-02

**Authors:** Amy M. Schwartz, Kiersten J. Kugeler, Christina A. Nelson, Grace E. Marx, Alison F. Hinckley

**Affiliations:** Centers for Disease Control and Prevention, Fort Collins, Colorado, USA

**Keywords:** Lyme disease, public health surveillance, incidence, vector-borne infections, zoonoses, United States, bacteria, ticks, Borrelia

## Abstract

We evaluated MarketScan, a large commercial insurance claims database, for its potential use as a stable and consistent source of information on Lyme disease diagnoses in the United States. The age, sex, and geographic composition of the enrolled population during 2010–2018 remained proportionally stable, despite fluctuations in the number of enrollees. Annual incidence of Lyme disease diagnoses per 100,000 enrollees ranged from 49 to 88, ≈6–8 times higher than that observed for cases reported through notifiable disease surveillance. Age and sex distributions among Lyme disease diagnoses in MarketScan were similar to those of cases reported through surveillance, but proportionally more diagnoses occurred outside of peak summer months, among female enrollees, and outside high-incidence states. Misdiagnoses, particularly in low-incidence states, may account for some of the observed epidemiologic differences. Commercial claims provide a stable data source to monitor trends in Lyme disease diagnoses, but certain important characteristics warrant further investigation.

Lyme disease is caused by infection with certain *Borrelia* spirochetes and transmitted to humans by *Ixodes* ticks (*1*). It is the most commonly reported vectorborne disease in the United States, despite a highly focal geographic distribution (*1*,*2*). Most reported cases of Lyme disease occur in 14 states in the Northeast, mid-Atlantic, and upper Midwest, although the geographic area with elevated disease risk is expanding (*2*,*3*). Lyme disease affects persons of all ages, but incidence peaks in children and older adults, presumably due to behaviors that put persons of these age groups in more frequent contact with infected ticks (*2*).

Lyme disease has been a nationally notifiable condition in the United States since 1991. Healthcare providers report cases to state or local health authorities, who evaluate the information and transmit it to the Centers for Disease Control and Prevention (CDC) through the National Notifiable Diseases Surveillance System (NNDSS) (*4*). Lyme disease surveillance was designed to provide public health officials with data to monitor trends and inform decision making. However, as the frequency and geographic distribution of Lyme disease cases have grown, so too has the burden of conducting surveillance. Several high-incidence jurisdictions are pursuing alternative ways to reduce the associated human resource and fiscal burden of conducting Lyme disease surveillance (*5*–*7*). As more jurisdictions adopt alternative sampling, estimation, or triage methods, the comparability of information gained from notifiable disease surveillance decreases (*5*,*7*).

Alternative data sources are increasingly more accessible and could supplement our understanding of the epidemiology of Lyme disease (*6*). Although intended for billing purposes, insurance claims data have been used to describe the epidemiology of many types of conditions (*8*–*10*), including the frequency and characteristics of clinician-diagnosed Lyme disease, its geographic distribution, and risk factors for disseminated illness (*11*,*12*). We expand on prior work by Nelson et al. (*11*) to examine the reliability of commercial claims data as an annual source of data on Lyme disease diagnoses. Specifically, we evaluated the stability and representativeness of a single commercial claims database during 2010–2018, variability in characteristics of identified Lyme disease diagnoses, and comparability to data obtained through routine passive surveillance.

## Methods

### Data Sources

IBM Watson Health MarketScan Commercial Claims and Encounters (CCAE) databases contain deidentified health encounter information on >25 million US residents <65 years of age who receive employer-sponsored health insurance, including early retirees and Consolidated Omnibus Budget Reconciliation Act (COBRA) continuees, and their dependents. Consistent with the methods described in Nelson et al., we restricted the MarketScan population to persons who had insurance coverage for an entire calendar year and who had the potential for associated pharmaceutical claims data to more accurately convey annual rates of coded Lyme disease diagnoses (*11*). State of primary beneficiary residence was used as a proxy for patient residence.

### Evaluation of the Stability and Representativeness of MarketScan

We evaluated characteristics of the insured population included in the MarketScan CCAE databases each year during 2010–2018 to define overall and annual population volume, composition, and representativeness with respect to sex, age, and geographic distribution. To evaluate the representativeness of the MarketScan population as compared with the US population <65 years of age, we used annual data from the US Census Bureau Vintage 2018 population estimates (*13*). To assess geographic representation given the focal nature of Lyme disease, we grouped states in geographic categories of Lyme disease endemicity in accordance with a recent Lyme disease surveillance summary (*2*). Connecticut, Delaware, Maine, Maryland, Massachusetts, Minnesota, New Hampshire, New Jersey, New York, Pennsylvania, Rhode Island, Wisconsin, Vermont, and Virginia were classified as high-incidence states. Illinois, Indiana, Iowa, Kentucky, Michigan, North Carolina, North Dakota, Ohio, South Dakota, Tennessee, West Virginia, and the District of Columbia all shared >1 border with a high-incidence state or were located between areas of high-incidence and thus were classified as neighboring states. All other states were classified as low-incidence for the purpose of this analysis.

### Identification of Lyme Disease Diagnoses in MarketScan

International Classification of Diseases (ICD) diagnosis codes are included in inpatient and outpatient healthcare encounter records in MarketScan; <15 diagnosis codes are included in each inpatient record and <4 diagnosis codes are included in each outpatient record. ICD-9-CM codes from the ICD, 9th Revision, Clinical Modification (ICD-9-CM), were used before October 2015; after this date, coding specialists were required to use codes from the ICD, 10th Revision, Clinical Modification (ICD-10-CM), in the United States (*14*).

For this analysis, we defined an outpatient Lyme disease diagnosis as the first outpatient healthcare encounter record per calendar year with a diagnosis code for Lyme disease (ICD-9-CM code 088.81 or ICD-10-CM code A69.2x) and a prescription for >7 days of treatment with an antimicrobial drug appropriate for Lyme disease and filled within +30 days of the encounter date. This approach was highly similar to the previous effort by Nelson et al. but with the necessary addition of ICD-10-CM codes (*11*) (Appendix). We defined an inpatient Lyme disease diagnosis as a hospitalization record that contained a principal diagnosis code for Lyme disease, or a principal diagnosis code of a documented objective clinical manifestation of Lyme disease or a tickborne disease transmitted by the same vector (e.g., babesiosis) and a secondary diagnosis code for Lyme disease in the same record (Appendix). We included 1 Lyme disease diagnosis per person per calendar year; we used the earliest date of service on which all criteria were met as proxy for illness onset date for analysis of seasonality.

### Comparison of Lyme Disease Diagnoses in MarketScan with Cases Reported through Public Health Surveillance

Lyme disease cases are classified and reported by states according to the Council of State and Territorial Epidemiologists surveillance case definition in effect during the year of report (*4*). For our analysis, we used confirmed and probable cases among those <65 years of age reported to CDC during 2010–2018. We compared Lyme disease diagnoses as identified in MarketScan to those of cases reported through national public health surveillance with respect to incidence, seasonality, sex, age, and geographic distributions.

### Statistical Comparisons

To compare sex, age, and geographic distributions between the MarketScan population and the US population (2014 estimates) and compare distributions of select characteristics of Lyme disease diagnoses versus cases identified through public health surveillance, we used both χ^2^ tests and Cramer’s V values, an approach similar to that used by Nelson et al. (*11*). Whereas χ^2^ tests are influenced by large cell sizes, Cramer’s V is not and provides more insight into the magnitude of similarity between the 2 populations (*11*). We considered Cramer’s V values <0.1 to indicate minimal to no difference between distributions because low values of Cramer’s V suggest a high goodness-of-fit. We used SAS software version 9.4 (SAS Institute, https://www.sas.com) for data management and analysis.

## Results

**Figure 1:**
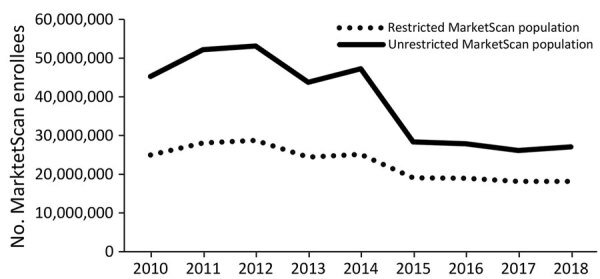
Annual restricted and unrestricted MarketScan database enrollment population by year, United States, 2010–2018. The restricted MarketScan population was limited to enrollees with insurance coverage for an entire calendar year, with the potential for pharmaceutical claims data, and a primary beneficiary residing in the United States (excluding territories when possible).

Health insurance claims from a mean of 39,004,340 enrollees were captured in the MarketScan database annually from 2010–2018; the lowest annual total was 26,146,275 persons in 2017 and the highest 53,131,420 in 2012. When restricting this population to persons enrolled for an entire calendar year and with available prescription data, a mean of 22,869,944 persons met these criteria annually, with the lowest total of 18,166,082 persons in 2017 and the highest 28,747,962 in 2012 (Figure 1). Demographic characteristics of the restricted and unrestricted MarketScan populations did not notably differ (data not shown), although the number of persons in the restricted population was more stable over time (Figure 1). Henceforth, the MarketScan population figures we cite here reflect the restricted population.

### Stability and Representativeness of MarketScan as an Annual Data Source

**Figure 2:**
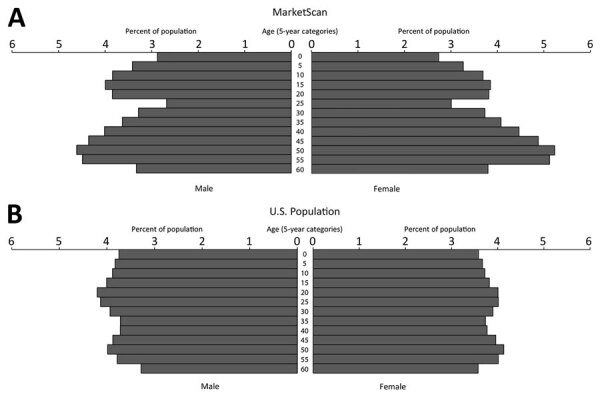
Population composition comparison of MarketScan enrollees (A) and US population (B) by age group and sex, United States, 2010–2018.

Age, sex, and geographic distributions of the MarketScan population were qualitatively stable during the study period, showing <8% proportional variation among years. The annual median age in MarketScan was 35−36 years; median age of the US population <65 years was lower, 32 years. Overall, MarketScan contained a smaller proportion of children 0–9 years of age and adults 25–29 years of age and a larger proportion of adults 40–59 years of age compared with the US population (p<0.0001 by χ^2^ test; Cramer’s V = 0.042); however, the low Cramer’s V value suggests comparability in the age distributions (Figure 2). Female enrollees were slightly overrepresented in the MarketScan population during the study period (median 51.7% female, annual range 51.3%–51.9% female) compared with the US population <65 years of age (49.8%–49.9% female) (p<0.0001 by χ^2^ test; Cramer’s V = 0.009); however, the very low Cramer’s V value suggests this difference is unlikely to be meaningful.

Overall, the regional representation in the MarketScan population based upon geographic categories of Lyme disease endemicity differed slightly from that of the US population (p<0.0001 by χ^2^ test; Cramer’s V = 0.026); however, the Cramer’s V value suggests lack of a substantial difference between these geographic distributions. An average of 25.6% of the US population resided in high-incidence states for Lyme disease, and 23.7% of the MarketScan population (range 20.1%–28.1%) resided in high-incidence states. Whereas an average of 52.8% of the US population resided in low-incidence states during the study period, 51.0% (range 47.1%–54.0%) of the MarketScan population resided in low-incidence states.

### Characteristics of Lyme Disease Diagnoses in MarketScan vs. Cases Reported through Surveillance

**Figure 3:**
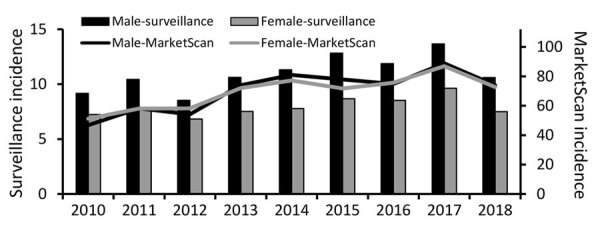
Incidence of patients with Lyme disease diagnoses in MarketScan database versus cases found by surveillance, by sex, United States, 2010–2018. Incidence was calculated as diagnoses/100,000 enrollees in MarketScan or cases/100,000 population among each subcategory. Scales for the primary and secondary y axes differ substantially to underscore sex-related incidence patterns but do not permit direct comparison of the magnitude of Lyme disease incidence between systems.

We identified 140,281 MarketScan enrollees with Lyme disease diagnoses during 2010–2018, of whom 1.2% were hospitalized. The minimum in a year was 12,256 enrollees in 2010; the maximum, 19,880 in 2014. Median incidence of Lyme disease diagnoses during 2010–2018 was 73.3/100,000 enrollees; annual incidence ranged from a low of 49.1/100,000 enrollees in 2010 to a high of 87.9/100,000 enrollees in 2017 (Table 1). By comparison, median annual incidence of Lyme disease (among those <65 years of age) according to surveillance was 9.3 cases/100,000 population; incidence ranged from 7.9/100,000 population in 2012 to 11.8/100,000 population in 2017 (Table 1). Annual variability in incidence of Lyme disease diagnoses in MarketScan tracked with a similar trajectory to the annual variability in surveillance data (Figure 3).

**Table 1:** Characteristics of Lyme disease diagnoses in MarketScan database versus national surveillance, United States, 2010–2018*

Category	2010	2011	2012	2013	2014	2015	2016	2017	2018
Overall incidence									
MarketScan	49.1	58.2	56.2	73.0	79.0	74.7	75.2	87.9	73.3
Surveillance	8.4	9.3	7.9	9.2	9.7	10.9	10.4	11.8	9.2
Incidence among male enrollees									
MarketScan	46.8	58.2	54.1	74.0	81.0	77.9	74.8	88.9	73.9
Surveillance	9.2	10.4	8.5	10.6	11.3	12.8	11.9	13.7	10.6
Incidence among female enrollees									
MarketScan	51.2	58.1	58.1	72.0	77.2	71.7	75.6	86.9	72.7
Surveillance	7.2	7.7	6.8	7.5	7.8	8.7	8.5	9.6	7.5
Seasonality, peak month (% of total occurring during May−August)									
MarketScan	June (53.0)	June (55.2)	June (52.0)	July (59.4)	July (60.1)	July (60.5)	June (53.6)	July (57.9)	June (56.9)
Surveillance	June (68.8)	June (71.4)	June (64.6)	July (73.7)	July (72.8)	July (74.0)	June (69.2)	July (71.0)	June (66.0)

*Incidence calculated as diagnoses/100,000 enrollees in MarketScan or cases/100,000 population among each subcategory.

#### Seasonality

The seasonal distribution of Lyme disease diagnoses peaked in the summer months, as it does for cases reported through surveillance (Table 1). Nevertheless, proportionally fewer coded diagnoses occurred during the historically higher incidence season for Lyme disease of May–August (57%) than among cases reported through surveillance (70%) (p<0.0001 by χ^2^ test; Cramer’s V = 0.142) (Table 1).

#### Sex and Age Distributions

Median annual incidence of Lyme disease diagnoses among male enrollees was 74.0 (range 46.8–88.9) diagnoses/100,000 population; median annual incidence among female enrollees was similar at 72.0 (range 51.2–86.9) cases/100,000 enrollees. In comparison, median incidence of cases among the male population according to surveillance was 10.6 (range 8.5–13.7) diagnoses/100,000 enrollees; median incidence among the female population was generally lower at 7.7 (range 6.8–9.6) cases/100,000 population (Table 1; Figure 3). Proportionally more diagnoses in MarketScan were among female patients compared with cases identified through surveillance (p<0.0001 by χ^2^ test; Cramer’s V= 0.095).

The sex and age distributions of Lyme disease diagnoses showed similar patterns across the years under study (Appendix Figure). Although both MarketScan and surveillance data display a bimodal age distribution with incidence peaks among children 5–9 years of age and adults >50 years of age, the peak among adults was more pronounced for diagnoses in MarketScan (p<0.0001 by χ^2^ test; Cramer’s V = 0.126) (Appendix Figure).

#### Geographic Distribution

State of residence was available for 94.9% of Lyme disease diagnoses captured in MarketScan during 2010–2018. Of these, ≈80.5% (range 76.6%–83.6%) were from high-incidence states. Although that figure represents most diagnoses, it was smaller than the 93.2% of cases reported from high-incidence states through surveillance (p<0.0001 by χ^2^ test; Cramer’s V = 0.216). Median annual incidence of Lyme disease diagnoses per 100,000 enrollees in MarketScan in high-incidence states was 242.8 (range 190.8–264.3); in neighboring states, 21.5 (range 14.8–32.0); and in low-incidence states, 15.0 (range 11.7–19.9). Median annual incidence per 100,000 population of Lyme disease according to surveillance in high-incidence states was 34.3 (range 28.7–43.0); in neighboring states, 2.1 (range 1.2–3.4); and in low-incidence states, 0.3 (range 0.3–0.5).

A smaller proportion of coded diagnoses identified in MarketScan occurred during the peak months of May–August compared with cases reported from surveillance across each geographic region (p<0.0001 by χ^2^ test and Cramer’s V = 0.1–0.2 for all 3 regional comparisons). Among diagnoses identified in MarketScan, a higher proportion from high-incidence states (59%) occurred during the summer compared with diagnoses from neighboring (53%) and low-incidence states (42%) (p<0.0001 by χ^2^ test; Cramer’s V = 0.113) (Table 2).

**Table 2:** Characteristics of Lyme disease diagnoses in MarketScan and reported cases in national surveillance by geographic category of Lyme disease endemicity, United States, 2010–2018*

Characteristic	Geographic category of Lyme disease endemicity							
	High-incidence states			Neighboring states			Low-incidence states	
	MarketScan	Surveillance		MarketScan	Surveillance		MarketScan	Surveillance
No. cases	107,125	220,320		10,891	11,435		15,117	4,627
% M	50.8	58.5		41.9	57.1		36.6	46.6
% F	49.2	41.5		58.1	42.9		63.4	53.4
Incidence among male enrollees/population	237.9	40.4		18.5	2.5		11.3	0.3
Incidence among female enrollees/population	220.5	28.5		24.1	1.9		18.2	0.4
No. (%) occurring during May−August	63,251 (59)	112,660 (70)		5,792 (53)	6,631 (73)		6,291 (42)	2,172 (62)
% Change in incidence rate, 2010–2018	19.5	7.4		88.9	177.0		48.0	14.7

*Incidence calculated as diagnoses/100,000 enrollees in MarketScan or cases/100,000 population among each subcategory.

**Figure 4:**
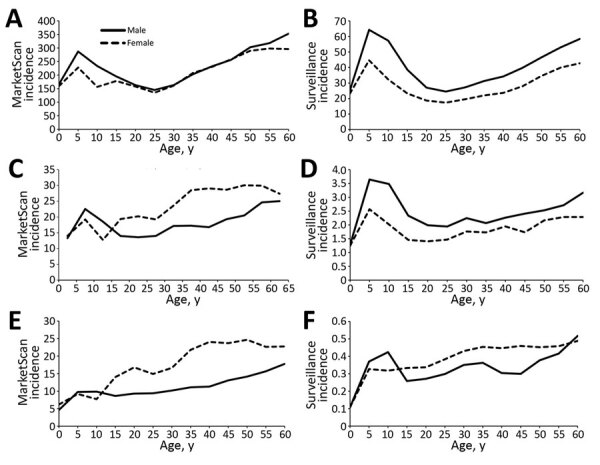
Lyme disease incidence by age group and sex in MarketScan enrollees (A, C, E) and from surveillance (B, D, F) by geographic category of Lyme disease endemicity (A– B, high-incidence states; C–D, neighboring states; E–F, low-incidence states), United States, 2010–2018. Incidence was calculated as diagnoses/100,000 enrollees in MarketScan or cases/100,000 population among each subcategory. Scales for each y-axis differ substantially to underscore overall age-related incidence patterns but do not permit direct comparison of the magnitude of Lyme disease between systems or geographic categories.

In both MarketScan and surveillance data, patient age distributions by sex differed across high-incidence, neighboring, and low-incidence states (Figure 4). Male patients accounted for a greater proportion of diagnoses in high-incidence states (50.8%) than in neighboring (41.9%) and low-incidence (36.6%) states (Table 2). Among high-incidence states, the peak incidence of diagnoses was among children 5–9 years and adults >50 years of age, and incidence was elevated among male enrollees across all ages, similar to trends seen in surveillance (Figure 4). In the neighboring states, a peak in incidence among male children was apparent in both MarketScan and surveillance data; however, disproportionately more diagnoses were among female enrollees >15 years of age. In low-incidence states, we observed no obvious trend by age and sex, and overall, the rate of diagnoses among female enrollees was higher than for male enrollees across most age groups (Figure 4).

During 2010–2018, the overall rate of coded Lyme disease diagnoses as identified in MarketScan increased 20% in high-incidence states and 48% in low-incidence states and nearly doubled (89%) in neighboring states (Table 2). Lyme disease incidence according to surveillance during this period increased 7% in high-incidence states and 15% in low-incidence states and more than doubled (177%) in neighboring states.

## Discussion

MarketScan, containing data on >25 million persons annually, is one of the largest sources of health insurance claims data currently available for US residents. We evaluated this database for its potential to serve as a stable source of data on Lyme disease diagnoses. Despite annual fluctuations in the size of the covered population and its restriction to commercially insured persons <65 years of age, the MarketScan population was demographically similar to the US population. Temporal trends observed in MarketScan data were similar to those observed in surveillance data, although the relative rate of diagnoses was substantially higher than that of reported cases. The median incidence of coded Lyme disease diagnoses in MarketScan was 73/100,000 enrollees during 2010–2018, ≈62% higher than the 45/100,000 enrollees observed in MarketScan during 2005–2010 (*11*), a temporal increase in Lyme diagnoses comparable to that reported in another insurance claims–based analysis (*15*). In addition, both the rate of Lyme disease diagnoses based on insurance claims and disease incidence as reported through surveillance increased substantially in states neighboring traditionally high-incidence states, a pattern consistent with ongoing geographic expansion of Lyme disease. Lyme disease diagnoses increased at a slower pace in traditionally high-incidence areas, a possible indication that disease risk is becoming more stable in these states. From this analysis, we conclude that MarketScan can serve as a stable source of data for annual evaluation of epidemiologic trends among Lyme disease diagnoses.

The higher incidence observed for Lyme disease diagnoses in MarketScan compared with cases identified through public health surveillance can be explained in large part by underreporting (*16*,*17*). However, variability in seasonal, demographic, and geographic distributions between data from these 2 systems suggest that some proportion of Lyme disease diagnoses captured through MarketScan are the result of misdiagnosis or miscoding. A larger proportion of Lyme disease diagnoses in MarketScan occurred outside of peak summer months, in female enrollees, and outside high-incidence states, compared with cases reported through surveillance. These characteristics may reflect the inclusion of other medical conditions for which Lyme disease may be considered in a differential diagnosis (*18*–*21*). In addition, given our objective of evaluating MarketScan for use on an annual, routine basis, our analysis treats each year independently. Individual patients could meet our designated criteria in multiple years, and consequently, a portion of identified diagnoses may actually reflect retreatment for a nonincident condition. Nevertheless, 91% of persons were diagnosed only once during the 9-year time frame.

In a recent evaluation of >1,200 persons referred for tertiary evaluation for Lyme disease in a high-incidence area, nearly three quarters lacked clinical or laboratory evidence of *Borrelia burgdorferi* infection; the majority of these persons referred with a diagnosis of Lyme disease were female and experiencing a long duration of constitutional symptoms (*18*). Given the relative scarcity of infected host-seeking ticks in low-incidence areas, the potential for locally acquired Lyme disease is often very low (*22*,*23*). Moreover, this low likelihood of Lyme disease translates to an increased probability of false-positive test results and, in turn, misdiagnoses for both humans and animals (*24*–*27*). In both MarketScan and surveillance data, the epidemiologic characteristics of Lyme disease differ between low- and high-incidence regions, consistent with proportionally more misdiagnoses in low-incidence states (*2*,*25*,*28*,*29*). Similarly, in a previous evaluation of Lyme disease cases reported through surveillance from low-incidence states (*25*), epidemiologic characteristics of cases with recent travel to high-incidence areas differed from those cases lacking reported travel. Further study would be helpful to understand which conditions, signs, or symptoms may be commonly mistaken for Lyme disease in these areas.

We used a Lyme disease−specific ICD code combined with appropriate antimicrobial treatment as a proxy measure for clinical diagnosis, a measure that is subject to limitations. Comprehensive laboratory data were not available for the majority of MarketScan enrollees and were not used to identify or rule out Lyme disease diagnoses. ICD codes are primarily used by medical institutions for billing, not for health studies, and practices are known to vary by coder and facility (*30*). We attempted to minimize use of rule-out codes by marrying temporally relevant treatment information, but some persons counted as Lyme disease diagnoses may not have received treatment for presumptive Lyme disease, but for another condition for which a similar antimicrobial therapy may be appropriate. Conversely, prior research suggests that Lyme disease−specific ICD codes are often omitted from medical records of patients with Lyme disease (*16*,*31*,*32*). Thus, the diagnoses summarized here using disease-specific codes likely reflect a fraction of all Lyme disease diagnoses and are therefore not comprehensive, even within the MarketScan database (*32*). Additional efforts analyzing coding patterns can be employed to create generalizable estimates regarding the incidence of clinician-diagnosed Lyme disease, which cannot be construed from these data alone (*11*,*33*). Despite statistical tests that indicated significant differences in the distributions of sex, age, and geographic representation between the MarketScan population and the US population, very low Cramer’s V values together suggest minimal differences in these distributions. However, the MarketScan CCAE databases do not contain information on uninsured persons, adults >65 years of age, or members of the military; consequently, these data are not entirely representative of the US population. Exploration of Medicare and Medicaid data may provide more insight into patterns of Lyme disease in populations not reflected in this analysis.

As access to electronic data sources for health-related information increases, more diverse data can be queried to more comprehensively inform the epidemiology of Lyme disease. However, when using novel data sources, the volume, stability, and representativeness must be considered before drawing inference. We evaluated the potential for 1 commercial health insurance claims database, MarketScan, to provide reliable information on an annual basis about the epidemiology of Lyme disease diagnoses. Despite limitations in generalizability of the data source and incompleteness of use of Lyme disease−specific codes, MarketScan provided a stable source of data for Lyme disease diagnoses that is comparable across years and could serve as a resource-efficient adjunct to surveillance. Although Lyme disease diagnoses identified from claims data are not supported by the robust evidence of infection required for surveillance reporting, they are a consistent indicator of trends in the healthcare system. In addition, the sheer volume of data available through MarketScan provides potential for new insights into the epidemiology of Lyme disease diagnoses in the United States.

Appendix Additional information about the use of commercial claims data for evaluating trends in Lyme disease diagnoses in the United States.
