# Supplementary material for: Use of Commercial Claims Data for Evaluating Trends in Lyme Disease Diagnoses, United States, 2010–2018
Source: Emerg Infect Dis. 2021 Feb;27(2):499–507. doi: 10.3201/eid2702.202728 (PMC7853566; doi:10.3201/eid2702.202728)
Supplement: Appendix — Additional information about the use of commercial claims data for evaluating trends in Lyme disease diagnoses in the United States. [file 20-2728-Techapp-s1.pdf]

Article DOI: https://doi.org/10.3201/eid2702.202728

Use of Commercial Claims Data for Evaluating Trends in Lyme Disease Diagnoses, United States, 2010-2018

Appendix

Appendix Table. Diagnosis codes for Lyme disease case identification in the MarketScan database*

Manifestation	ICD-9 codes	ICD-10 codes
Lyme disease	088.81	A69.20, A69.21, A69.22, A69.23, A69.29
Facial palsy	351.0, 351.8, 351.9, 352.6, 781.94, 951.4, 951.8, 951.9	G51.0, G51.8, G51.9, G52.7, G52.8, G52.9, G53, R29.810, S04.50XA, S04.51XA, S04.52XA, S04.891A, S04.892A, S04.899A, S04.9XXA
Lyme carditis	420.0, 420.90, 420.91, 420.99, 422.0, 422.90, 422.91, 422.92, 422.93, 422.99, 429.0, 427.81, 426.0, 426.10, 426.11, 426.12, 426.13, 426.2, 426.3, 426.4, 426.50, 426.51, 426.52, 426.53, 426.54, 426.6	I30.1, I30.9, I30.0, I30.8, I41, I40.9, I40.0, I40.1, I40.8, I51.4, I49.5,, I44.2, I44.30, I44.0, I44.1, I44.4, I44.5, I44.60, I44.69, I44.7, I45.0, I45.10, I45.19, I44.30, I44.39, I45.4, I45.2, I45.3, I45.5, I45.89, I45.9
Meningitis	322.9, 320.7, 320.82, 320.89, 320.9,322.0	G03.9, G01, G00.9, G00.8, G04.2, G03.0
Arthritis	711.00, 711.01, 711.02, 711.03, 711.04, 711.05, 711.06, 711.07, 711.08, 711.09, 711.40,711.41, 711.42, 711.43, 711.44, 711.45, 711.46, 711.47, 711.48, 711.49, 711.80, 711.81, 711.82, 711.83, 711.84, 711.85, 711.86, 711.87, 711.88, 711.89, 711.90, 711.91, 711.92, 711.93, 711.94, 711.95, 711.95, 711.97, 711.98, 711.99, 716.60, 716.61, 716.62, 716.63, 716.64, 716.65, 716.66, 716.67, 716.68, 719.00, 719.01,719.02,719.03,719.04,719.0, 719.06, 719.07, 719.08, 719.09	M00.80, M00.9, M00.811, M00.812, M00.819, M00.821, M00.822, M00.829, M00.831, M00.832, M00.839, M00.841, M00.842, M00.849, M00.851, M00.852, M00.859, M00.861, M00.862, M00.869, M00.871, M00.872, M00.879, M00.88, M00.89, M01.X0, M02.80, M01.X11, M01.X12, M01.X19, M02.811, M02.812, M02.819, M01.X21, M01.X22, M01.X29, M02.821, M02.822, M02.829, M01.X31, M01.X32, M01.X39, M02.831, M02.832, M02.839, M01.X41, M01.X42, M01.X49, M02.841, M02.842, M02.849, M01.X51, M01.X52, M01.X59, M02.851, M02.852, M02.859, M01.X61, M01.X62, M01.X69, M02.861, M02.862, M02.869, M01.X71, M01.X72, M01.X79, M02.871, M02.872, M02.879, M01.X8, M02.88, M01.X9, M02.89, M01.X21, M01.X22, M01.X29, M02.822, M01.X32, M01.X39, M01.X51, M01.X52, M02.851, M01.X61, M01.X69, M02.861, M02.869, M01.X71, M01.X29, M01.X39, M01.X49, M01.X59, M01.X79, M13.10, M13.111, M13.112, M13.119, M13.121, M13.122, M13.129, M13.131, M13.132, M13.139, M13.141, M13.142, M13.149, M13.151, M13.152, M13.159, M13.161, M13.162, M13.169, M13.171, M13.172, M13.179, M25.40, M25.411, M25.412, M25.419, M25.421, M25.422, M25.429, M25.431, M25.432, M25.439, M25.441, M25.442, M25.449, M25.451, M25.452, M25.459, M25.461, M25.462, M25.469, M25.471, M25.472, M25.473, M25.474, M25.475, M25.476, M25.48
Tickborne diseases transmitted by same vector	088.82, 082.40, 082.41,082.49	B60.0, A77.40, A77.41, A77.49

*Antimicrobial drugs used for establishment of inclusion criteria for outpatient events can be found in Nelson et al. (*11*)

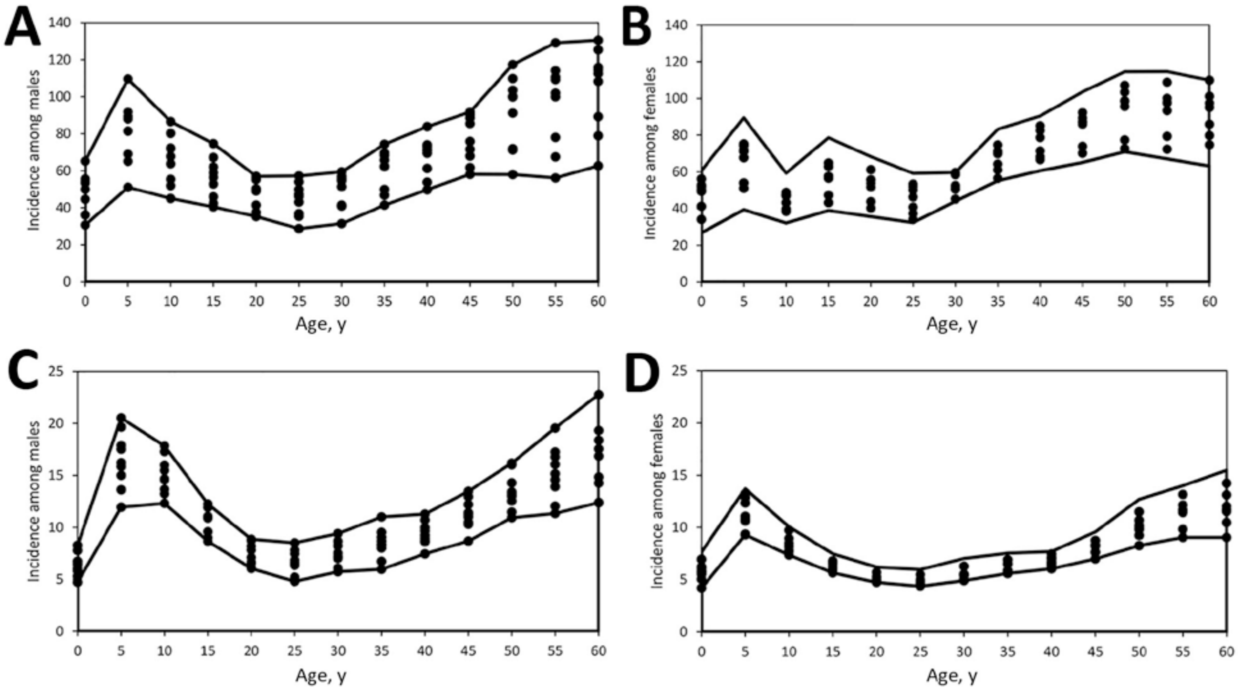

Appendix Figure. Annual incidence by age and sex according to MarketScan databases (A,B) and surveillance (C,D), United States, 2010–2018. Incidence calculated as diagnoses/100,000 enrollees in MarketScan or cases/100,000 population among each subcategory. Each dot represents the Lyme disease incidence for each age and sex category for each year during 2010–2018.
